# Degradation of Biodegradable Nonwoven Mulches in the Winter Period

**DOI:** 10.3390/polym16162279

**Published:** 2024-08-11

**Authors:** Dragana Kopitar, Paula Marasovic

**Affiliations:** Department of Textile Design and Management, Faculty of Textile Technology, University of Zagreb, Prilaz Baruna Filipovica 28a, 10000 Zagreb, Croatia; paula.marasovic@ttf.unizg.hr

**Keywords:** cellulose fibres, PLA fibres, nonwoven mulch blends, biodegradability, soil temperature and humidity, soil content

## Abstract

An open field experiment from November 2022 to May 2023 in Croatia, which is characterized by a continental humid climate, evaluated nonwoven mulches made from viscose, jute, and hemp fibres blended with PLA fibres. The blends of viscose and jute fibres (90:10, 80:20, and 70:30 ratios) were produced using mechanical web formation on cards with needle punching for bonding webs. Additionally, hemp fibres were blended with PLA fibres in a ratio of 80:20. Winter conditions caused significant structural changes in the mulches, including shrinkage, increased mass per unit area, thickness, and reduced air permeability. The amount of PLA fibre in the nonwoven mulch blends significantly affected nonwoven fabric structure change during exposure to winter conditions. After 180 days, the breaking force of all mulches increased by 30% to 277%. The soil beneath jute and hemp mulches maintained higher temperatures and moisture levels compared to viscose mulches. Soil organic carbon content varied with fibre type and was higher under jute and hemp mulches. K_2_O content was significantly higher in soils covered by mulches. All mulches effectively suppressed weeds. The experiment results showed that the newly produced nonwoven mulches could replace the conventional agro foil. Results also suggest that choosing biodegradable nonwoven mulches produced from fibres obtained from natural and renewable sources can influence soil fertility and the availability of nutrients, ultimately affecting plant growth and agricultural productivity.

## 1. Introduction

The rise in environmental standards for technological processes, difficulties in sourcing materials with desired properties, and advancements in agricultural technology are creating new opportunities for nonwoven products made from natural fibres and renewable cellulose sources [1]. Utilizing natural fibres in agrotextile production offers environmental advantages due to their biodegradability and renewability. Fibres like wool, cotton, palm leaves, flax, jute, and coir exhibit favourable mechanical properties and moisture retention, particularly suitable for agricultural applications like mulching. However, their adoption is hindered by higher costs, shorter lifespans compared to plastics, limited weathering resistance, and lower anti-microbial performance. Further research is crucial to understand the degradation rates and performance of agrotextiles made from natural fibres to become a viable alternative to plastics in agrotextiles [2].

Researchers have undertaken various initiatives to enhance these parameters. Saha et al. conducted a study to prolong the lifetime of jute-based geotextiles using non-hazardous, nontoxic, plant-based ingredients. The study shows that chemically treating jute fabrics leads to transesterification of cellulose chain hydroxyl groups increasing jute fibre crystallinity and resistance to degradation by three to five times [3,4].

Another strategy involves developing agrotextiles with improved anti-microbial activity, such as a hemp structure loaded with microcapsules for enhanced resistance to microorganisms. Agrotextiles were produced by wet laid technology, while microcapsules were prepared by a co-extrusion/gelling method with alginate as shell and oregano oil as core material. The microcapsules’ release of oregano oil was controlled and provided good antimicrobial activity compared to nonwoven fabrics that were not treated with microcapsules [5].

Investigations of recycled nonwoven agrotextile degradation under laboratory conditions during three months of exposure in a QUV device showed a decrease in mechanical properties and thermal stability [1]. The nonwoven fabric blends gave insight into the degradation of natural and synthetic fibres under accelerated weathering conditions confirming the faster rate of natural fibres degradation.

Biodegradation due to microorganisms is also evaluated, assessing the performance of woven agrotextiles buried in the soil and enzymatic hydrolysis with cellulose. Impregnation with Ag/TiO_2_ nanoparticles hindered the biodegradation of cotton and cotton/PET fabrics. Results confirm the complicated biodegradation of textile wastes and environmental impact (strongly dependent on the temperature and humidity of the soil) implying that metal and metal oxide nanoparticles originating from textile wastes in the terrestrial environment must be explored to a larger extent [6].

The exploration of recycled nonwoven fabrics for application in agriculture, namely cotton waste mulching fabric, showed good mechanical properties, light transmittance, and excellent degradation characteristics making it an eco-friendly and cost-effective material for agricultural use [7].

Researchers are actively working on enhancing the mechanical properties, weathering resistance, and lifespan of mulches and studying their degradation under diverse conditions. Current research on the biodegradation of polylactic acid (PLA) nonwoven fabric blends focuses on combinations with polyhydroxyalkanoate (PHA) or polyhydroxybutyrate (PHB) biopolymers, where investigations are focused on the influence of exposure in compost-enriched soil and laboratory-simulated weathering conditions. Accelerated biodegradation in soil, driven by specific microbial communities, underscores the complex relationship between polymer blends, environmental conditions, and microbial activity. Additionally, environmental conditions significantly influence PLA polymer degradation. Nonwoven melt-blown mulches made from bio-based polymers such as PLA and PLA/PHA blends and exposed to soil conditions degraded after 30 weeks. Melt-blown mulches, especially from PLA/PHA blends, exhibit a loss of tensile strength, which was influenced by factors such as soil moisture, soil temperature, and mulch composition [8,9,10,11,12].

Studies on PLA-based mulches reveal significant tensile strength loss, validating the first stage of biodegradation. The tensile strength loss of melt-blown nonwoven mulches is a crucial indicator of the biodegradation process, reflecting mulch weakening and deterioration. Environmental conditions and physical-chemical properties influence the rate and extent of mulch biodegradation, where loss of tensile strength is a valuable measure to observe and analyse the biodegradation process of nonwoven melt-blown mulches [13].

Incorporating cellulose fibres in PLA-based blends has been explored for improving the mechanical performance and stability of materials. Studies on jute/PLA composites show promising mechanical qualities and thermal stability of the material, supporting the need for international standards in agro-fibre manufacturing [14,15,16,17].

Agro foil mulching impacts soil moisture and temperature, whereas colourless agro foil has a pronounced effect on soil water content. During winter, agro foil mitigates spring drought, conserves water, and raises the temperature, stimulating early potato growth and increasing yields [18]. Additionally, mulching with agro foil reduces evaporation, enhances water use efficiency, boosts aboveground wheat biomass, and augments grain yield, demonstrating its significance in combating soil salinity, conserving water, and promoting efficient water utilization for plant growth [19].

Mulching with nonwoven agrotextiles, specifically jute mulches, during the winter season also showed a positive impact on soil health, moisture, microbial population, and nutrient content, at the same time promoting the growth and productivity of crops like broccoli harvested in spring as well [20].

For employing mulches for diverse purposes like weed suppression or maintenance of a desired microclimate, it is crucial to note that the seasonal temperatures will strongly influence mulch performance. Temperature significantly impacts mulch biodegradability, effectiveness, and soil property levels as microbial breakdown of organic materials occurs differently, depending on environmental factors like oxygen availability and temperature.

The composition and abundance of microorganisms (including fungi and bacteria), enzymatic activity, oxygen levels, and salt concentrations of soil play pivotal roles in determining the rate and extent of mulch biodegradation across various field conditions [21,22]. Nonwoven mulches influence soil temperature, consequently influencing the metabolic rates, growth, and activity of fungi and bacteria, influencing various soil processes and ecosystem functions. Different species of fungi and bacteria have their optimal temperature ranges for growth and activity. Soil temperature fluctuates seasonally, affecting microbial communities accordingly. During warmer seasons, microbial activity increases, leading to faster mulch decomposition rates and soil nutrient cycling. In contrast, colder temperatures during winter months can slow down microbial activity [23].

Comprehensive studies of nonwoven mulches produced by the mechanical process on a card and bonded with needle punching, from cellulose derivatives and PLA biopolymer fibres showed the degradation of mulches from cellulose derivatives after 6 months, depending on the type of fibres [24,25,26]. After two years of field study, the degradation of mulches made of PLA fibres is only slightly noticeable. The mulches made from their blends showed that the addition of 20% PLA fibres significantly affects the maintenance of the structure over time, which was manifested through a slower decrease in their properties (surface mass, thickness, tensile properties, air permeability).

The path to sustainable agrotextiles could lie in nonwoven mulch blends made from natural fibres and biodegradable PLA polymer. Challenges regarding natural fibre usage for nonwoven mulch production still exist, where advances in weather resistance and better performance could be improved by blending with PLA fibres. The hypothesis is that adding a certain percentage of PLA fibres to nonwoven mulches blended with viscose, jute, and hemp fibres can regulate the decomposition time of mulches used in the winter period for crops that are planted in autumn and harvested in the spring/winter period.

Viscose fibres are derived from cellulose, which is sourced from plant materials. The production process for soluble cellulose is environmentally harmful, requiring more reagents and water compared to natural fibre production from bast crops. Despite its environmental impact, the worldwide production of man-made cellulosic fibres in 2021 amounts to 7.2 million tonnes of production volume, with viscose as the most important man-made cellulosic fibre holding 80% of the market share with a production volume of around 5.8 million tonnes in 2021 [27]. There is a great potential to recycle viscose textile materials to produce nonwoven fabrics for mulching applications. This approach would reduce the amount of viscose in landfills and create new mulching products from recycled materials. Due to its natural biodegradation and absorption properties, viscose mulches could replace conventional plastic films which by degradation pollute the earth with plastic, i.e., are harmful to the environment.

The mulch blends with PLA fibres would positively affect the soil quality, temperature, and humidity during the biodegradation, while simultaneously suppressing weeds, meeting the goals of the industry’s commitment to a greener and more sustainable future. The properties and performance of the mulches could be compared to conventional plastic agro foil, which would make possible agro foil replacement with environmentally friendly polymeric materials.

Therefore, this study analyses the biodegradation of nonwoven mulch blends made from cellulose derivate and different ratios of PLA fibre in the winter season, that is, the degradation of mulches installed in the fall and removed from the field in late spring. The impact of nonwoven mulches on soil quality and their ability to suppress weeds was determined and compared to uncovered soil and conventional agricultural film. The effect of nonwoven mulches on soil temperature and humidity was evaluated, contributing to a better understanding of agricultural season interdependence and the effectiveness of nonwoven mulches.

## 2. Materials and Methods

### 2.1. Materials

**Figure 1:**
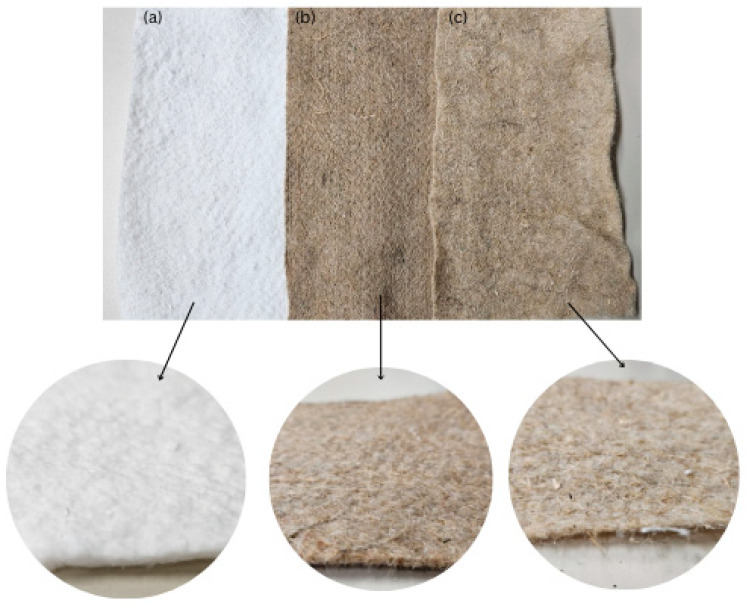
The nonwoven mulch blends with 20% of PLA fibres and (**a**) 80% of viscose, (**b**) 80% of jute, (**c**) 80% of hemp fibres.

Seven nonwoven mulch blends were produced from PLA fibres (derived from corn starch with a fineness of 6.84 dtex and a length of 64 mm supplied by NatureWorks BV, Plymouth, MN, USA) in ratios of 90:10, 80:20, and 70:30 with jute (31.02 dtex) and viscose (1.78 dtex, 40 mm) fibres supplied by Derotex (Table 1 and Table 2, Figure 1). The hemp fibres (58.54 dtex) were blended with PLA fibres only in a ratio of 80:20.

The bale openers opened the fibres from the bales of PLA fibres and particular cellulose derivates (jute, viscose, and hemp). The fibres were transported from the bale opener by air in multi-hopper systems where two types of fibres were blended in the desired ratio. Final blending was performed on the card where the web is formed. The web from the card was laid down on cross lappers and bonded by a needle-punching process. The nonwoven mulches were produced with the same production parameters in the nominal mulch mass per unit area of 300 g m^−2^ [25].

In the experiment were included a commercially available low-density polyethylene agro foil (Gerovit, Serbia; thickness of 20 µm, 28.17 g m^−2^) and a control field to compare biodegradation and performance of produced nonwoven mulches.

**Figure 2:**
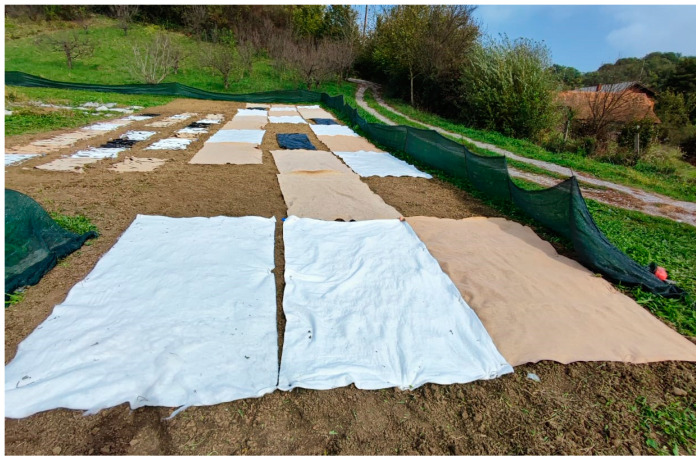
The winter field experiment with laid nonwoven mulch blends.

The nonwoven mulches and agro foil, dimensions 1.5 m × 1 m (1.5 m^2^), were placed on the soil in a random-block design of three replication plots, including the control field (Figure 2).

The field study took place in a humid continental climate (Dfa) according to Köppen–Geiger’s classification at Donji Laduč, Croatia (45°53′ N, 15°44′ E). Initiated in November 2022, the experiment concluded with the collection of the final nonwoven mulch replication plot in May 2023. Replication plots of nonwoven mulches and agro foil were removed after 60, 120, and 180 days of exposure. Weekly recordings of soil temperature and moisture were taken beneath the mulches. The degradation of the mulches was evaluated through physical-mechanical tests following each removal from the experimental site.

### 2.2. Methods

The air temperature and humidity data during the experiment were supplied by the hydro-meteorological station closest to the mulch exposure site. Soil moisture was measured at a depth of 15 cm using a PMS-714 soil moisture meter from Lutron Electronic Enterprise Co., Ltd., Taipei, Taiwan. At the same depth, soil temperature was measured with a LabTherm XL bi-metal dial thermometer (Dostmann electronic GmbH, Wertheim-Reicholzheim, Germany) featuring a waterproof stainless-steel probe.

The biodegradation of the nonwoven mulches was periodically evaluated based on their physical-mechanical properties as follows:

- Mass per unit area was determined according to ISO 9073-1:2023 [28] where five test specimens, each of 50,000 mm^2^ an area, were weighed in the standard atmosphere and expressed in g m^−2^;
- Nonwoven mulch thickness was tested according to ISO 9073-2:1995 for normal nonwovens (Method A) on 10 test pieces, each having an area larger than 2500 mm^2^ applying a pressure of 1 kPa [29];
- The air permeability of nonwoven fabric was measured on an Air Tronic device, Mesdan S.p.A., Puegnago Del Garda BS, Italy according to the ISO 9073-15:2007 standard on five circular specimens [30]. A flow rate of 10 litres per minute was used, regulating airflow until the desired pressure drops of 100 Pa were achieved, using a circular test area of 10 cm^2^;
- The mulches’ breaking force and elongation were determined according to the ISO 9073-3:2023 standard on wide strips [31]. Five samples per each nonwoven fabric production direction (MD, machine direction, and CD, cross-machine direction) of 200 mm width, using wide clamps for nonwoven textiles and with a distance between clamps of 200 mm, were tested on a Tenso Lab 5000, Mesdan S.p.A, Puegnago Del Garda BS, Italy tensile tester at a constant speed of 100 mm/min.

At the beginning and end of the experiment (after 180 days), soil samples were taken from beneath each mulch type and the control field using a cylindrical agrochemical probe at a depth of 0–30 cm. The physicochemical properties of the soil beneath the nonwoven mulches and the control field were analysed according to standard protocols.

- The soil’s pH was tested following the ISO 10390:2005 standard [32];
- The organic carbon content was calculated based on the total humus value;
- Humus content was determined using the bichromate method, where soil organic matter was wet-oxidized with potassium dichromate;
- The total nitrogen content was measured using the Kjeldahl method;
- Plant-available P_2_O_5_ and K_2_O were extracted using the AL method, which involved extracting phosphorus and potassium from the soil with an ammonium lactate solution at a pH of 3.75.

After each removal of the nonwoven mulches replication plot (after 60, 120, and 180 days of exposure), weeds grown through the mulches were collected, dried to an absolutely dry sample and then weighed on an analytical balance. The weed mass in the control field was considered 100% weeding. The weeding percentages for each mulch and agro foil were then calculated and expressed concerning the weed mass of the control field.

**Figure 3:**
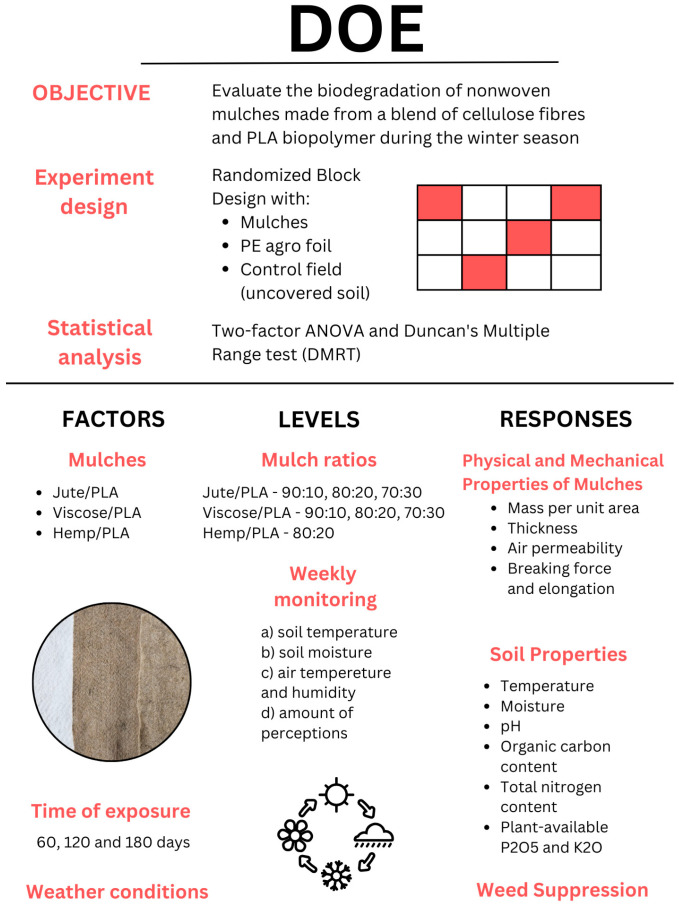
Design of the experiment of the investigation of biodegradable nonwoven mulches’ degradation in the winter period.

For a better understanding of the experiment design, influencing factors (mulches, exposure time and weather conditions), levels (mulch blends ratio, weekly monitoring of environmental conditions) and responses (results of weediness, mulches, and soil properties) of the experiment are presented in Figure 3.

## 3. Results and Discussion

The performance and degradation of nonwoven mulches made of viscose, jute, and hemp fibres in blends with PLA fibres (10%, 20%, and 30%) in a nominal mass per unit area of 300 g m^−2^ during the winter field experiment were investigated and compared with conventional agro foil. The samples’ labelling is presented in Table 2.

Nonwoven mulches mass per unit area, thickness, and air permeability, before exposure to field conditions (0 days) and after 60, 120, and 180 days of exposure with statistical indicators are presented in Table 3.

The mass per unit area of all nonwoven mulches increases. As the percentage of PLA fibres in nonwoven mulch blends increases, the increase in mulch masses due to environmental conditions decreases. The exception is viscose mulch with 30% PLA fibres, which has the highest mass increase of 56.7%. Comparing the jute and hemp nonwoven mulches with the same PLA fibre percentage (20%) shows a significantly lower mass increase for the hemp nonwoven blend. The reason could be explained as an interaction between the coarse stem fibres and the finer PLA fibre in the nonwoven structures.

The change in nonwoven mulch thickness and air permeability after 180 days of exposure is not consistent, i.e., for some blends they decreased (thickness up to 24.3%; air permeability up to 33.6%), while for some blends they increased (thickness up to 7.78%; air permeability up to 5.5%). According to the results, there was a significant change in the structure (mass per unit area, thickness, air permeability) of nonwoven fabrics due to environmental conditions. As a result of the shrinkage in the nonwoven mulches, an increase in mass per unit area occurs; consequently, mulch thickness increases and air permeability decreases. Besides shrinkage, the residue of impurities (earth particles, branches, leaves) could not be cleaned from the mulch structures (after collecting mulches from the field and drying) without additional damage. In contrast to nonwoven fabrics produced by extrusion processes (spun-bond, melt-blown), nonwoven fabrics produced on a card and bonded by needling have a layered structure (laying a certain number of the webs on a cross-lapper depending on the mass per unit area). Web layers consist of fibres, where the space between the crossing fibres of the web forms air-filled pores. During exposure to the field conditions in the layered structures of mulches, different plant and soil impurities enter the air-filled pores and layers of fibrous webs, consequently increasing mass per unit area and thickness and thus reducing air permeability [24]. In addition to all of the above, over time there has been a partial decomposition of the fibre in the mulches, which, together with the appearance of the residue of impurities and fabric shrinkage, gives results that are not easily explained due to their inconsistency. Because of all the above, monitoring mulch degradation through mass, thickness, and air permeability change is impossible for nonwoven fabrics produced on cards and bonded by needling.

Unlike the nonwoven mulch structure composed of fibrous layers, where the space between the crossing fibres forms pores that allow air and water vapour passage, PE agro foil is impermeable. For this reason, agro foil has not been tested for air permeability.

**Figure 4:**
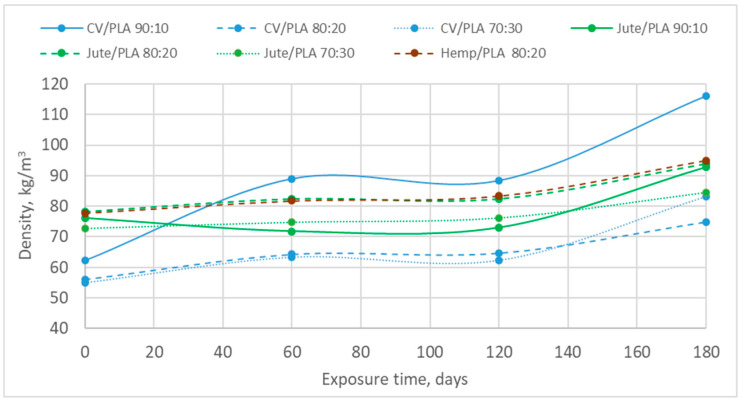
Nonwoven mulches’ density during winter field exposure.

To determine the alteration in the structure (density) of the nonwoven mulch due to field exposure, its density was calculated using the mass per unit area and thickness of the initial sample and samples collected after 60, 120, and 180 days of weathering. The density of the nonwoven fabric is the mass per unit volume of the nonwoven fabric (kg/m^3^), which is calculated from the measured weight per unit area (kg/m^2^) divided by the measured thickness of the fabric (m) for certain days of exposure (Figure 4, Table 4).

The density of viscose-blended mulches with a proportion of 10% of PLA fibres has a significantly higher increase compared to mulches with the addition of 20 and 30% of PLA fibres. Viscose mulches blended with 20% and 30% of PLA fibres show a smaller density change and a similar trend during exposure time. Mulch blends made of bast fibres, jute and hemp, and 20% PLA fibres have an almost identical change in density during the exposure time.

The density of all nonwoven mulches increased after 180 days of exposure to field conditions as the result of the previously explained structural changes, i.e., its shrinkage (Table 4). The greatest density increase was observed for mulches made of viscose and PLA nonwoven mulch blends, where the mulch blend with the lowest percentage of PLA fibres has a density change of 86.2%. Since the technological parameters of mulch production were the same for all mulches, same as an environmental condition during the period of mulches exposure, it can be concluded that mulch with the highest percentage of finer viscose fibres (1.78 dtex), regarding PLA fibres (6.8 dtex), packed more closely together during structure shrinkage resulting in a denser fabric. It should be considered that the mulch density was calculated from the mass per unit area and thickness results, which were affected by the previously explained parameters, i.e., mulch shrinkage and impurity residues in the layered structure. For the same reason, there were slight increases and decreases in mulch density during 180 days of exposure [25,26].

The increase in density of jute and PLA fibre mulch blends, after 180 days of exposure, has an increase in density of up to 21.9%. In mulches made of jute fibres, the influence of the percentage of PLA fibres in mulch blends is visible, i.e., with an increase in the proportion of PLA fibres in nonwoven mulch blends, the density decreases. The results confirm that an increase in the proportion of finer PLA fibres (6.8 dtex) in mulch blends with jute fibres (31.0 dtex) increases mulch density after 180 days of exposure to environmental conditions. The density of hemp and PLA fibre mulch blend is higher but approximately equal to the density of jute mulch of the same proportion of PLA fibres (80:20).

The different changes in the density of nonwoven mulches due to exposure to external influences were influenced by the parameters of the fibres themselves, i.e., the fineness and length of the fibres as well as the type of fibres. The nonwoven mulches produced by coarser and longer fibres (hemp and jute) had less nonwoven mulch density change, which is probably related to the structure change due to field conditions (shrinkage). The shrinkage of viscose mulch blends was up to 6% in MD and 0.6% in CD, jute mulch blends up to 4.7% in MD and 1% in CD, and hemp mulch shrank in MD up to 3.7% and in CD up to 1%. The phenomenon of mulch shrinkage was also observed in previous research [26].

**Figure 5:**
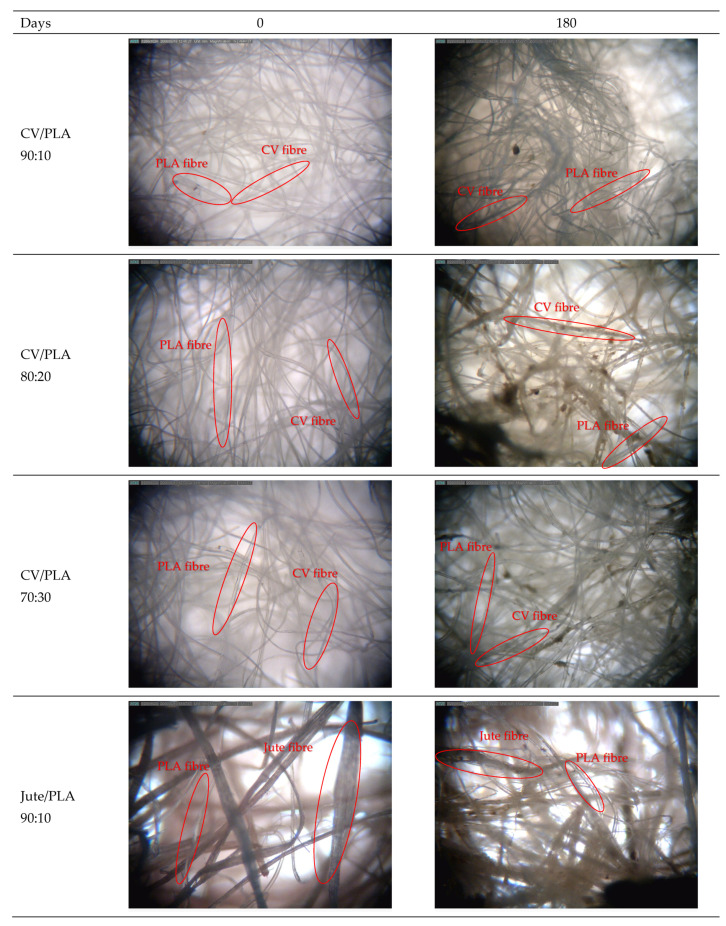

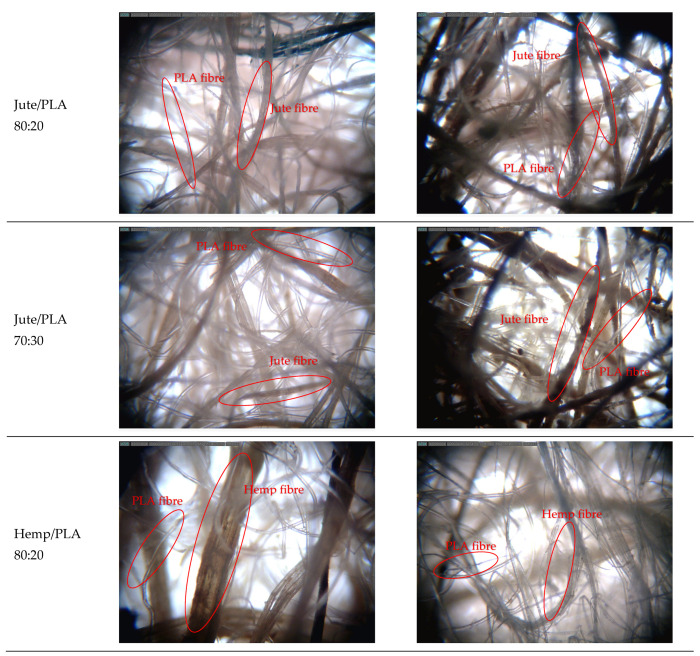
Microscopic images of unexposed nonwoven mulch blends and after 180 days of field exposure; magnification ×40.

Microscopic images of mulches made of viscose and PLA fibres show that the fibres have retained their shape and length but changed their density (Figure 5). In viscose blends, a significant structural change is visible, i.e., fibres come close to each other creating a compact structure, which was noted through the significant density increase regarding jute and hemp mulch blends. In the images of all nonwoven mulches after 180 days of exposure, the residue of impurities between fibres is visible. On nonwoven jute-blended mulches, a certain amount of soil was layered on the jute fibres while the PLA fibres remained clean.

Likely, the less compact structure of jute and hemp nonwoven mulch compared to viscose/PLA blends after 180 days of exposure may be attributed to variations in fibre fineness among jute, hemp, and PLA. This is evident in the lower mass per unit area and density increase observed in hemp and jute mulch blends in comparison to viscose mulches. It could be assumed that the different changes in the structures of nonwoven mulch blends after 180 days of exposure were influenced by the fibre parameters, respectively, fineness and the type of fibres [25,26].

**Figure 6:**
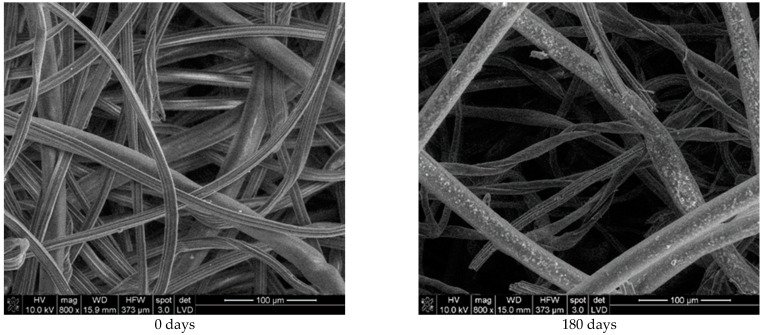
SEM images (×800) of viscose and PLA fibres from unexposed nonwoven mulches (0 days) and mulches exposed for 180 days to environmental conditions.

**Figure 7:**
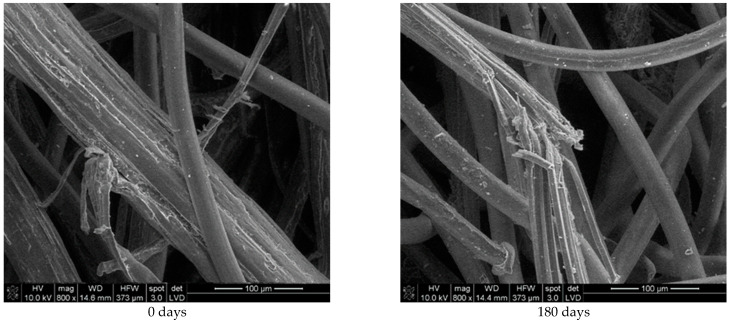
SEM images (×800) of jute and PLA fibres from unexposed nonwoven mulches (0 days) and mulches exposed for 180 days to environmental conditions.

**Figure 8:**
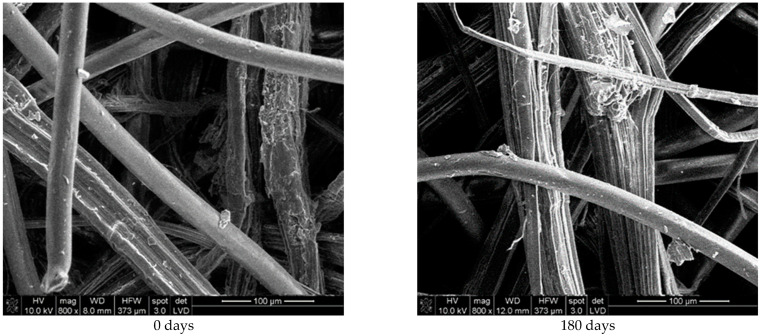
SEM images (×800) of hemp and PLA fibres from unexposed nonwoven mulches (0 days) and mulches exposed for 180 days to environmental conditions.

To obtain better insight into fibre degradation, SEM images of the 80:20 nonwoven mulch blends were made (Figure 5, Figure 6, Figure 7 and Figure 8). The viscose and PLA fibres for the unexposed sample appear relatively smooth and well-defined. Differences between finer viscose fibres (1.78 dtex) and coarser PLA fibres (6.84 dtex) are visible. The overall structure seems uniform, with distinct separations between the individual fibres. The fibres are randomly oriented, forming a loose network with overlapping regions, typical for nonwoven fabrics.

The viscose fibres after 180 days of exposure show noticeable signs of degradation. The fibres appear less smooth and more irregular, with signs of surface erosion and roughening compared to the initial state. Some viscose fibres seem to have started breaking down or becoming more brittle. The PLA fibres from unexposed mulch displayed a smooth and clean surface. After 180 days of environmental exposure, the SEM image shows the emergence of roughness and the presence of small pores on the PLA fibre surface. Viscose fibres have undergone more changes than PLA fibres. The fibre changes within the mulches could be due to environmental exposure and microbial activity in the soil on which the mulches were laid down.

Unexposed jute/PLA nonwoven mulch blends show a smooth PLA fibre surface (Figure 7). The surface of jute fibres appears rough with longitudinal striations or ridges. These striations are due to the microfibrillar structure of the fibre. After 180 days of exposure, jute fibres from mulch show surface damage, roughness, and prominent cracks compared to the unexposed sample. The microorganisms from the soil ate the outer protective layer of jute (pectin) whereby elementary jute fibres became visible. PLA fibres exhibit little change, similar to the change in PLA fibres in viscose/PLA nonwoven mulch blends.

Hemp fibres are composed of individual fibres closely packed together in bundles. The junctions and overlaps of hemp fibres are visible, showing the cohesion within the bundles (Figure 8). After 180 days of exposure, hemp fibres start to break down through a degradation process revealing free elementary fibres of which hemp fibres are made. PLA fibres show a lower degree of fibre surface change, like the PLA fibres in viscose and jute mulch blends.

**Figure 9:**
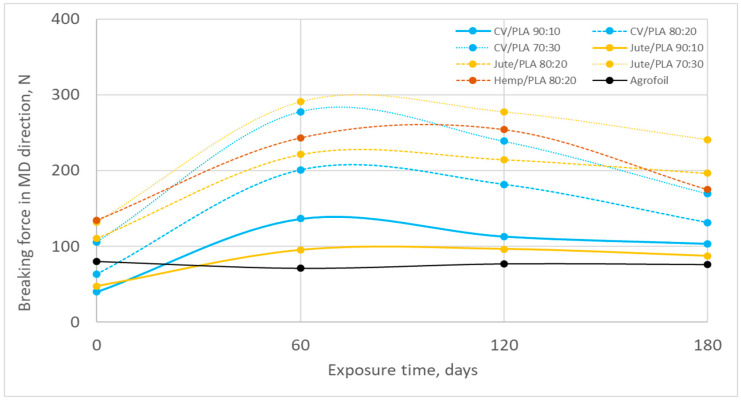
Breaking force in MD during winter field exposure.

**Figure 10:**
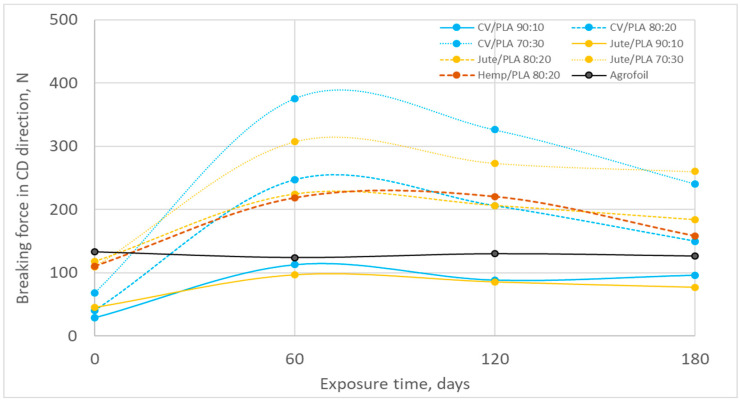
Breaking force in CD during winter field exposure.

**Figure 11:**
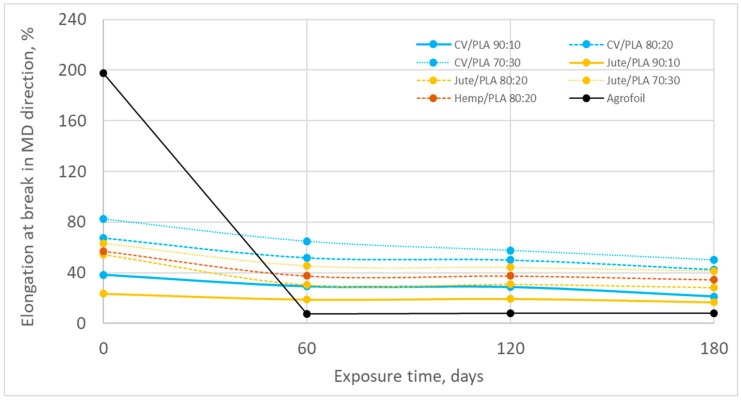
The elongation at break in MD during winter field exposure.

**Figure 12:**
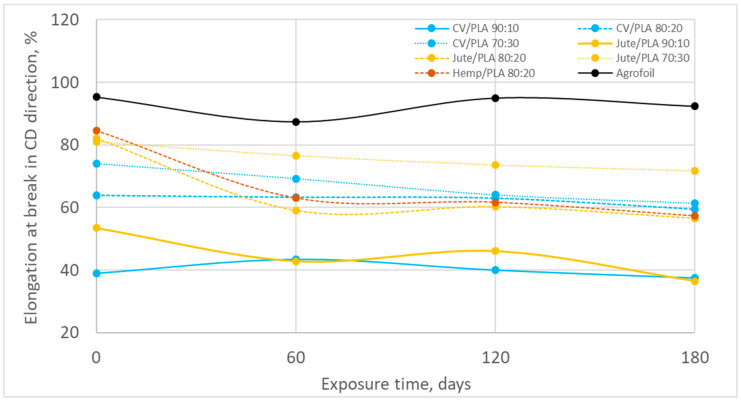
The elongation at break in CD during winter field exposure.

The breaking force in the machine direction and cross-machine direction and their change (%) during winter field exposure are presented in Figure 9, Figure 10, Figure 11 and Figure 12 and Table 5, Table 6, Table 7, Table 8, Table 9, Table 10 and Table 11. Unexposed mulch blends made of jute fibres have a higher breaking force than viscose mulch blends in both production directions (MD, CD) comparing mulch blends with the same percentage of PLA fibres. Although the breaking force of PLA fibres is lower than the breaking force of viscose, jute, and hemp fibres, increasing the amount of PLA fibres in nonwoven mulch blends significantly increases the breaking force of almost all mulch blends in both production directions [24]. As the PLA fibres are finer and less rigid compared to jute and hemp fibres, by increasing the percentage of PLA fibres in blends better fibre entangling during the needle-punching process was provided. Better fibre entangling results in higher cohesion between the fibres and, consequently, higher mulch tenacity. Due to the nonwoven structure that contains fibres of significantly different fineness, length, and surface characteristics, a more compact and closer interconnection of two different fibre types in webs occurred. During nonwoven fabric production on cards and bonding by needle punching, dispersion, and arrangement of two different types of fibres within the webs, their mutual interaction and ability to entangle and form a cohesive structure affected the breaking force [25,26].

After 180 days of exposure to environmental conditions, the breaking force in the MD (from 30% to 160%) and CD (from 43% to 277%) of all nonwoven mulches increased. It coincides with the previously obtained mass, thickness, and density increase and air permeability decrease, i.e., the shrinkage of the nonwoven mulch structure (Figure 9 and Figure 10, Table 5, Table 6 and Table 7).

For an easier and better understanding of significant changes in breaking force during exposure time, Table 4 and Table 6 show the changes in breaking force of nonwoven mulches in MD (Table 5) and CD (Table 7) in percentages. The breaking force change for nonwoven mulches is expressed relative to the breaking force of the previous exposure period in the percentages, i.e., the breaking force changes of mulch between unexposed (0 days) and after 60 days, the percentage change after 120 days is relative to the breaking force after 60 days, and the percentage change after 180 days is relative to the breaking force after 120 days. The breaking force change in percentage terms between unexposed nonwoven mulch (0 days) compared to mulch after 180 days of exposure is given in the last columns of Table 5 and Table 7.

On the 60th day of winter exposure, the breaking force increased for all nonwoven mulches (Figure 9 and Figure 10). Previous research has shown that the structure of nonwoven mulches produced from cellulose and PLA fibres changes significantly in the first 60 days of exposure. The air humidity and temperature, rainfall, and soil influence the cellulose derivative fibres in the nonwoven mulches where fibres swell and come closer to each other, decreasing the spaces between the fibres filled with air (nonwoven fabric pores) and thus creating higher-density nonwoven structures [25,26]. After 60 days of exposure, the breaking force of nonwoven mulch blends in both production directions tends to decrease until the end of the experiment.

Considering the percentage of PLA fibres, a change in breaking force of nonwoven mulch blends, in both fabric production directions, during winter exposure to field conditions was not observed (Table 5 and Table 7).

The reason is due to the complexity and nature of fibre orientation in the cross-laid nonwoven fabrics that ultimately influence the anisotropic property of the nonwoven fabric and consequently the breaking force [33]. Since the ratio of breaking force in the cross -machine direction (CD) and machine direction (MD) reflects the nature of fibre orientation in the nonwoven fabric, it can be concluded that fibres in mulch blends during the period of exposure to field conditions, besides swelling and coming closer to each other, change orientation in the fabric to a certain extent (Table 6). A CD/MD ratio equal to 1 shows minimum anisotropy of fibres in nonwoven mulches, i.e., isotropic structure.

Considering the CD/MD ratio, after 180 days of the experiment, the fibres in viscose mulch blends become more aligned to the CD of the fabric as the percentage of PLA fibres increases.

Contrary to viscose mulches, in the jute mulch blends the fibres become more aligned to the MD as the PLA percentage and time of exposure increases. The jute mulch blend with the highest percentage of PLA fibres (30%) is an exception. It is evident that even unexposed mulch already has significantly different fibre orientations, considering the same blends with a lower percentage of PLA fibres, i.e., the structure is more orientated in the MD due to the interaction of the two significantly different fibres, meaning better fibre entangling during the needle-punching process. Therefore, a trend of breaking force increase in the CD of unexposed jute blends with an increase in the PLA fibre percentage is evident, until 30% of PLA fibres are blended. The breaking force in the CD direction of the 90:10 jute mulch blend was 44.70 N, for 80:20 it was 118.12 N, and for 70:30 it dropped to 109.28 N, meaning the fibre orientation changes.

Due to environmental conditions after 180 days of the experiment, the orientation of jute mulch blend with 30% PLA fibres became more orientated to CD, unlike blends with a lower PLA percentage.

Considering the many variables that affect the structure of nonwoven mulch blends with the influence of field conditions, making a conclusion about mulch degradation based on the change in breaking force is complex.

In contrast to nonwoven mulches, the breaking force of conventional agro foil in both production directions (MD, CD) after 180 days decreased equally, by 5%.

Two-factor ANOVA without replication was performed to determine if differences in the breaking force of mulches in MD and CD were significant. An ANOVA test of the breaking force showed that there are statistical differences between all mulches, except for the 80:20 jute and hemp nonwoven mulch blends in the MD and 80:20 of jute, hemp, and viscose nonwoven mulch blends in the CD (Table 8). It can be concluded that the addition of 10–30% of PLA fibres into mulches made of cellulose fibres (viscose, hemp, and jute) after 180 days of exposure to field conditions changes the structure in such a way that the breaking force of all mulches is higher than before the field exposure. In addition, the percentage share of PLA fibres in the nonwoven mulch blends significantly affects nonwoven fabric structure change during 180 days of exposure to winter conditions.

The elongation at break of all nonwoven mulches after 180 days of exposure to environmental conditions decreased. Depending on the mulch composition and production direction, the decrease in elongation at break ranged from a minimum of 3.89% to a maximum of 48.28 (Figure 11 and Figure 12, Table 9 and Table 10). A higher elongation at break decrease in MD is visible.

Although the elongation at break of viscose fibres (16.38%) is significantly higher than PLA fibres (6.35%), with increasing PLA fibres in mulch blends the elongation at break does not decrease probably due to the lesser compactness of the structure visible through density of viscose mulch blends (Table 4, Figure 5) [24]. Less compactness means the fibre entangling is poorer and the dominancy of fibre slippage is evident.

The increase in elongation at break by blending more PLA fibres in jute mulch blends is due to lower breaking extension of jute fibres (3.4%) compared to PLA (6.35%) [24].

After exposure to the field conditions, elongation at break in the MD and CD fabric decreases (Figure 11 and Figure 12) leading to the conclusion of fibre straightening and breakage. The influence of the amount of PLA fibres in the CD is visible. The change in elongation at break of viscose mulch blends decreases by increasing the percentage of PLA fibres (Table 10) due to fibre slippage more than fibre breakage considering the breaking force changes and slower degradation of mulches produced by viscose and PLA fibres [25,26]. The contrary is obtained for jute mulch blends, where mulch blends with a higher percentage of jute fibres have a greater change in elongation of break (Table 10). It is evident that after 180 days a certain degradation of jute fibres occurs, where the change in the mulches’ elongation of break is due to fibre breakage. The more jute fibres in the mulch blends, the greater the change in elongation at break after field exposure.

The elongation at break of conventional agro foil in MD after 180 days is significant leading to the conclusion that agro foil become extremely fragile. In CD the change is minor, only 5%. The reason can be found in the orientation of the molecules of PE agro foil, which is primarily affected by the processes of extrusion and stretching during production.

Although an impact of PLA fibre percentage in the mulch blends or environmental conditions on elongation at break change was not determined, a two-factor ANOVA without replication was performed to determine if the differences in the elongation at break of mulches in MD and CD were significant (Table 11). An ANOVA test showed that there are statistical differences between elongation at break of all mulches, except for the 80:20 jute and hemp nonwoven mulch blends in the MD and CD.

The average soil temperature and moisture in the period from November 2022 to May 2023, with the associated average air temperature and air humidity, are presented in Table 12 and Table 13. Generally, during the winter experiment, soil temperatures under the nonwoven mulches follow the temperature trend under the foil and in the control field (Table 12). The soil temperature under mulches made from viscose fibres in blends with PLA fibres is lower than under nonwoven mulches made from jute and hemp fibres. The temperatures under the nonwoven mulches are higher than in the control field, except from 17 February to 24 March, when they are lower in the range of 0.3 to 0.9 °C. In that period, the soil temperatures under the nonwoven mulches were also lower, compared to the soil under the conventional agro foil. The influence of the different PLA fibre percentages of nonwoven mulches on the temperature of the soil under the mulches was not observed.

During the winter experiment, soil moisture under the mulches mainly follows the soil moisture trend under the foil and in the control field. The influence of the type of fibres or the percentage of PLA fibres in the nonwoven mulch blends on soil moisture was not observed. Soil moisture under the mulches is higher than in the control field, except from 17 November to 8 December, when the soil moisture is lower in the range of 0.9 to 2.4%.

A two-way ANOVA was conducted to assess the statistical significance of temperature and soil moisture under various mulches and in the control field during the winter period, November 2022 to May 2023. The ANOVA revealed significant differences between temperatures beneath mulches and on the control field. Therefore, Duncan’s new multiple range test (MRT) was performed (Table 14). In winter 2022/2023, the statistical differences in soil temperatures between viscose nonwoven mulch soil temperatures regarding nonwoven mulches and agro foil are obtained. The difference in soil temperature between winter mulches and agro foil is noted; meaning the temperature under agro foil is statistically significantly different than under the nonwoven mulch blends. There is a correlation between CV/PLA 80:20 mulches and nonwoven mulch blends regarding the soil temperature. There is no difference in the soil temperature between jute/PLA mulch blends, CV/PLA 90:10, and hemp/PLA mulch blends.

In the same period, a statistical difference in soil moisture beneath mulches and in the control field was not found (*p* = 0.3248). It is assumed that due to the significant winter precipitation and low soil temperature, the mulches absorbed the maximum amount of water, depending on the type of fibre, and remained constantly moist during exposure to external conditions, maintaining approximately the same soil moisture. For a more detailed conclusion, it is necessary to investigate the absorption characteristics of nonwoven mulches at certain temperatures.

The effect of mulches on soil chemical properties after 180 days of mulching is presented in Table 15. A decrease in soil solution pH (pH H_2_O) compared to soil solution pH before mulching was observed in all mulched treatments, i.e., the alkalinity of the soil is slightly reduced (from 7.85 to 7.71). Increasing the PLA percentage in viscose and jute nonwoven mulches decreases soil pH levels. The influence of PLA fibre percentage in mulches on the soil solution pH and the reserve acidity in the colloids (pH KCl) was not observed. Decreased soil pH could be explained by reduced evaporation when nonwoven mulches are used on the soil [34].

Since the nonwoven mulches reduced CO_2_ emission from soil respiration, part of CO_2_ can be transformed into carbonic acid. Also, CO_2_ fertilisation can promote plant growth by stimulating root development and increasing the exudation of organic acids [35]. It is logical to assume that with nonwoven mulch degradation, soil evaporation will increase, decreasing the effect of mulches on soil pH.

Soil organic carbon and total nitrogen significantly affect soil physical, chemical, and biological properties, which can affect crop productivity [36]. Considering the results (Table 15), it could be noticed that the percentage of soil total organic carbon before the experiment is higher than after the experiment, i.e., in soil on the control field and beneath all mulches. Generally, natural mulches provide habitat and food sources for soil microorganisms [37]. Microbial activity beneath the mulch layer accelerates the decomposition of organic matter, leading to the accumulation of organic carbon in the underlying soil [38]. Favourable conditions for microbial communities include moderate soil temperature fluctuations. Lower temperatures and higher wet soils over the winter probably lead to carbon losses through processes such as denitrification and methane emissions. The lowest organic carbon percentage was found in soil mulched with viscose nonwoven mulch blends with higher PLA fibre percentages (80:20 and 70:30). Soil covered with other mulches had a higher organic carbon content compared to soil on the control field after the experiment. The mulches probably act as soil insulation, maintaining relatively stable soil temperatures and microbial activity at a slower pace beneath the mulches [39].

The soil temperature under mulches made from viscose fibres in blends with PLA fibres is lower, especially with higher PLA fibres percentage, than under nonwoven mulches made from jute and hemp fibres, which provided the lowest organic carbon percentage. The highest organic carbon content was found in soil covered by hemp/PLA nonwoven mulch blend, where the mulch maintained the highest average temperature during the experiment. Several factors can influence the total carbon percentage in the soil beneath mulches of the same mass per unit area layered in the same soil composition, that is, mulch composition and soil microbial activity [40].

The total nitrogen on the control field before and after the experiment is almost the same (Table 15). Total nitrogen beneath all mulches is considerably higher. Considering the structure and tensile properties of nonwoven mulches during 180 days, where breaking force tended to decrease after 60 days, it looks like a certain rate of degradation of the fibres within nonwoven structure after 180 days is reflected in an increase in soil nitrogen content. Certain degradation of nonwoven mulches influences humus content, where soil covered by mulches with a lower percentage of PLA fibres contains a higher percentage of humus, which is higher than in soil covered by agro foil.

After the experiment, the content of P_2_O_5_ in the soil is lower beneath mulches and on the control field, except for soil covered by viscose/PLA mulch (90:10). The content of K_2_O available to plants is significantly higher in the soil after the experiment, especially in soils covered by nonwoven mulches. Previous investigations showed that K_2_O levels in soil significantly affected plant yield; hence, mulching with the proper biodegradable nonwoven mulch could improve yields of different plants [41,42]. The studies show that increasing potassium oxide levels in the soil significantly influenced the number, diameter, and weight of plants as well as total yield. The highest yields were obtained with higher levels of K_2_O application, with significant differences observed between the control group and the treatments. Hence, mulching with the proper biodegradable nonwoven mulch could improve the yields of different plants as the K_2_O levels in the soil increase. This highlights the importance of choosing a sustainable material that will control weeds and naturally increase K_2_O levels without the need for fertilizers.

The findings regarding soil composition indicate that the composition of fibres used in mulches affects their decomposition rates, thereby leading to varying effects on soil nutrient content, humus levels, and carbon levels. Selecting different types of fibres for nonwoven mulch production can influence soil fertility and the availability of nutrients, ultimately affecting plant growth and agricultural productivity.

The nonwoven mulches were successful in weed control compared to the weediness of the control field (Table 16).

The nonwoven mulches successfully suppressed weed growth. After 180 days of winter experiment, the maximum percentage of weeds grew up in the field covered by the jute/PLA 70:30 nonwoven mulch (3.2%) compared to the weediness of the control field. Mulches could be suitable for bulbs planted in late autumn, when the fruits are harvested in spring. For example, nonwoven mulches could prevent the development of weeds while creating favourable temperature conditions for the development of young onions in the early spring period. Conducting an open-field study is essential to evaluate the impact of newly developed nonwoven mulches on the growth, yield, and nutrient content of specific plant species.

## 4. Conclusions

Designed mulches, made from natural and regenerated cellulose blended with PLA fibres, were tested during the winter in the field to evaluate their impact on soil quality, weed suppression, and degradation. The mass per unit area of nonwoven mulch blends increased due to environmental exposure, while thickness changes varied among blends. All blends showed increased density, suggesting fibre parameters influenced structural changes such as shrinkage.

After 180 days, the breaking force of the mulches in both machine and cross -machine directions increased (up to 160.34%), particularly with higher cellulose fibre content in the nonwoven mulch blends. It could be concluded that adding 10 to 30% of PLA fibres in mulch blends made of cellulose fibres (viscose, hemp, and jute) after exposure to field conditions changes the structure significantly, which increases the breaking force of all mulches.

Soil temperature and humidity under the nonwoven mulches were consistent with trends observed under foil and control conditions, although statistical differences in soil temperature were noted compared to agro foil.

Soil alkalinity was slightly reduced beneath the mulches, with higher PLA percentages in viscose and jute blends lowering soil pH. Total organic carbon content decreased beneath all mulches, due to lower temperatures and higher wet soils over the winter, which led to carbon losses. Compared to the control field after the experiment, the favourable conditions for microbial communities beneath mulch blends (moderate soil temperature fluctuations) led to lesser carbon content losses.

After the experiment, total nitrogen was higher beneath all mulches compared to the control field, indicating fibre degradation. Humus content was higher under mulches with lower PLA percentages than under agro foil suggesting degradation of cellulose fibres.

The findings suggest mulch composition directly affects decomposition rates, influencing soil nutrient levels, humus content, and carbon levels, ultimately impacting plant growth and productivity. All tested nonwoven mulches were effective in weed control.

Overall, these new mulches show potential to replace conventional agro foil, providing benefits in degradation stability, soil quality, temperature and humidity maintenance, and weed control.

## 5. Future Recommendations

Based on the investigation findings, further research should include the impact of biodegradable nonwoven mulches on crops that are planted before winter and harvest in spring/summer such as spring onion or cabbage plants to understand how the degradation of the mulch may affect crops.

The presented research is limited by the humid continental climate region. Different climatic areas have a significant influence on the biodegradation of nonwoven mulches, and therefore research should be extended to different climatic regions to gain insight into their biodegradation and changes in their properties.

The need to collaborate with agricultural experts, environmental scientists, and industry stakeholders to further develop and promote the adoption of biodegradable nonwoven mulches as sustainable alternatives to traditional agro foil is emerging. It is important to assess the economic feasibility of replacing conventional agro foil with biodegradable nonwoven mulches on a larger scale to understand the cost-effectiveness and practicality of implementing this alternative in agriculture.

## Figures and Tables

**Table 1:** Mechanical properties of viscose, jute, hemp, and PLA fibres [24].

Fibre	Fibre Fineness, dtex	Fibre Lengths, mm	Elongation at Break, %	Fiber Tenacity (cN/tex)
Viscose	1.78	40	17.1	21.4
Jute	31.02	-	4.1	43.9
Hemp	58.54	-	5.9	62.5
PLA	6.84	64	7.1	17.9

**Table 2:** The nonwoven mulch labels.

Label	Raw Material Composition
CV/PLA 90:10	90% viscose fibres, 10% PLA fibres
CV/PLA 80:20	80% viscose fibres, 20% PLA fibres
CV/PLA 70:30	70% viscose fibres, 30% PLA fibres
Jute/PLA 90:10	90% jute fibres, 10% PLA fibres
Jute/PLA 80:20	80% jute fibres, 20% PLA fibres
Jute/PLA 70:30	70% jute fibres, 30% PLA fibres
Hemp/PLA 80:20	80% hemp fibres, 20% PLA fibres
Agro foil	conventional PE (polyethylene) agro foil

**Table 3:** Mass per unit area and thickness of nonwoven mulches after 180 days of field exposure.

Samples	Properties	Days			
		0	60	120	180
CV/PLA 90:10	Mass per unit area, g m^−2^	289.8	335.9	351.8	408.4
	σ, g m^−2^	0.7	3.3	1.6	2.5
	CV, %	3.3	13.9	6.3	8.7
	Thickness, mm	4.6514	3.7719	3.9779	3.5200
	σ, mm	0.2511	0.7750	0.3797	0.3595
	CV, %	5.3985	20.5469	9.5452	10.2142
	Air permeability, cm^3^/s/cm^2^	52.50	38.49	48.64	34.84
	σ, cm^3^/s/cm^2^	4.07	4.86	8.23	8.19
	CV, %	7.75	12.62	16.93	23.50
CV/PLA 80:20	Mass per unit area, g m^−2^	224.7	261.7	268.4	302.1
	σ, g m^−2^	0.2	1.2	1.1	2.4
	CV, %	1.5	6.4	6.0	11.6
	Thickness, mm	4.0015	4.0696	4.1481	4.0359
	σ, mm	0.1357	0.3563	0.3492	0.7228
	CV, %	3.3923	8.7557	8.4189	17.9090
	Air permeability, cm^3^/s/cm^2^	78.60	81.36	97.27	82.72
	σ, cm^3^/s/cm^2^	5.47	5.65	9.00	10.28
	CV, %	6.96	6.94	9.26	12.42
CV/PLA 70:30	Mass per unit area, g m^−2^	257.6	298.1	294.9	403.6
	σ, g m^−2^	0.4	2.0	2.1	5.7
	CV, %	2.2	9.4	10.1	20.2
	Thickness, mm	4.6739	4.7019	4.7242	4.8572
	σ, mm	0.2238	0.3969	0.4813	0.6053
	CV, %	4.7893	8.4405	10.1886	12.4625
	Air permeability, cm^3^/s/cm^2^	74.10	85.07	99.82	78.16
	σ, cm^3^/s/cm^2^	5.01	5.21	7.63	11.53
	CV, %	6.76	6.12	7.65	14.75
Jute/PLA 90:10	Mass per unit area, g m^−2^	277.7	290.6	293.8	364.9
	σ, g m^−2^	0.5	1.3	1.5	2.7
	CV, %	2.7	6.2	7.3	10.7
	Thickness, mm	3.6515	4.0465	4.0272	3.9357
	σ, mm	0.1264	0.2666	0.2212	0.6212
	CV, %	3.4614	6.5876	5.4938	15.7845
	Air permeability, cm^3^/s/cm^2^	186.24	205.79	240.15	141.16
	σ, cm^3^/s/cm^2^	28.98	41.60	44.51	26.75
	CV, %	15.56	20.22	18.53	18.95
Jute/PLA 80:20	Mass per unit area, g m^−2^	290.0	334.1	325.7	344.2
	σ, g m^−2^	0.5	3.9	3.1	5.1
	CV, %	2.3	16.8	13.6	21.0
	Thickness, mm	3.7068	4.0521	3.9527	3.6649
	σ, mm	0.2210	0.3256	0.2956	0.3371
	CV, %	5.9629	8.0362	7.4778	9.1990
	Air permeability, cm^3^/s/cm^2^	197.38	163.05	202.99	145.65
	σ, cm^3^/s/cm^2^	45.35	48.36	35.04	18.80
	CV, %	22.97	29.66	17.26	12.91
Jute/PLA 70:30	Mass per unit area, g m^−2^	265.0	284.1	281.4	313.6
	σ, g m^−2^	0.4	1.3	1.1	3.1
	CV, %	2.2	6.3	5.7	14.3
	Thickness, mm	3.6395	3.8007	3.6936	3.7170
	σ, mm	0.1149	0.1799	0.1545	0.2525
	CV, %	3.1570	4.7339	4.1823	6.7922
	Air permeability, cm^3^/s/cm^2^	180.38	178.32	221.55	146.53
	σ, cm^3^/s/cm^2^	18.76	21.35	22.19	16.46
	CV, %	10.40	11.97	10.02	11.24
Hemp/PLA 80:20	Mass per unit area, g m^−2^	317.1	310.9	318.7	348.8
	σ, g m^−2^	0.6	1.3	1.6	2.8
	CV, %	2.7	5.9	7.3	11.6
	Thickness, mm	4.0780	3.8024	3.8215	3.6693
	σ, mm	0.1299	0.1961	0.2362	0.2405
	CV, %	3.1847	5.1576	6.1812	6.5543
	Air permeability, cm^3^/s/cm^2^	196.40	243.99	255.75	197.89
	σ, cm^3^/s/cm^2^	26.94	51.14	47.95	41.18
	CV, %	13.72	20.96	18.75	20.81
Agro foil	Mass per unit area, g m^−2^	28.2	30.5	29.9	30.9
	σ, g m^−2^	0.0	0.1	0.1	0.1
	CV, %	1.8	4.2	2.6	2.5
	Thickness, mm	0.0670	0.1447	0.0730	0.0830
	σ, mm	0.0120	0.0636	0.0202	0.0329
	CV, %	17.8551	43.9084	27.7236	39.6271

Where: σ is standard deviation, CV is coefficient of variation.

**Table 4:** The density of mulches during field exposure (kg/m^3^) and change in density (%).

Samples		Days				Change in Density, %
		0	60	120	180	0–180
CV/PLA	90:10	62.3	89.0	88.4	116.0	86.2
CV/PLA	80:20	56.1	64.3	64.7	74.9	33.5
CV/PLA	70:30	55.1	63.4	62.4	83.1	50.8
Jute/PLA	90:10	76.1	71.8	73.0	92.7	21.8
Jute/PLA	80:20	78.2	82.4	82.4	93.9	20.1
Jute/PLA	70:30	72.8	74.8	76.2	84.4	15.9
Hemp/PLA	80:20	77.8	81.8	83.4	95.0	22.1
Agro foil		420.4	210.4	410.2	371.8	−11.6

**Table 5:** The breaking force change (%) of mulches in machine direction (MD) during winter field exposure.

Days	Breaking Force Change, %			
	0–60	60–120	120–180	0–180
CV/PLA 90:10	243	−17	−9	160
CV/PLA 80:20	218	−9	−28	108
CV/PLA 70:30	163	−14	−29	60
Jute/PLA 90:10	101	1	−9	84
Jute/PLA 80:20	100	−3	−8	77
Jute/PLA 70:30	121	−5	−13	83
Hemp/PLA 80:20	81	5	−31	30
Agro foil	−11	8	−1	−5

Where—denotes decrease.

**Table 6:** The breaking force in CD/MD ratio during winter field exposure.

Days	0	60	120	180
CV/PLA 90:10	0.7	0.8	0.8	0.9
CV/PLA 80:20	0.6	1.2	1.1	1.1
CV/PLA 70:30	0.6	1.4	1.4	1.4
Jute/PLA 90:10	0.9	1.0	0.9	0.9
Jute/PLA 80:20	1.1	1.0	1.0	0.9
Jute/PLA 70:30	0.8	1.1	1.0	1.1
Hemp/PLA 80:20	0.8	0.9	0.9	0.9
Agro foil	1.7	1.7	1.7	1.7

**Table 7:** The breaking force change (%) of mulches in cross-machine direction (CD) during winter field exposure.

Days	Breaking Force Change, %			
	0–60	60–120	120–180	0–180
CV/PLA 90:10	298	−22	9	239
CV/PLA 80:20	522	−17	−27	277
CV/PLA 70:30	451	−13	−26	253
Jute/PLA 90:10	115	−12	−10	71
Jute/PLA 80:20	90	−8	−11	56
Jute/PLA 70:30	181	−11	−5	138
Hemp/PLA 80:20	98	1	−28	43
Agro foil	−7	5	−3	−5

Where—denotes decrease.

**Table 8:** The statistical analysis of the mulches’ breaking force in MD and CD.

Samples Tested	*p*-Value
All nonwoven mulches and agro foil in MD	5.96 × 10^−9^
All nonwoven mulches in MD	3.53 × 10^−7^
All viscose nonwoven mulches in MD	2.17 × 10^−4^
All jute nonwoven mulches in MD	2.65 × 10^−3^
Viscose, jute, and hemp nonwoven mulch blends (80:20) in MD	8.83 × 10^−4^
Jute and hemp nonwoven mulch blends (80:20) in MD	0.31
All nonwoven mulches and agro foil in CD	3.00 × 10^−12^
All nonwoven mulches in CD	1.26 × 10^−10^
All viscose nonwoven mulches in CD	4.39 × 10^−6^
All jute nonwoven mulches in CD	2.46 × 10^−4^
Viscose, jute, and hemp nonwoven mulch blends (80:20) in CD	0.10
Jute and hemp nonwoven mulch blends (80:20) in CD	0.21

**Table 9:** The elongation at break change (%) of mulches in MD during winter field exposure.

Days	0–60	60–120	120–180	0–180
CV/PLA 90:10	−24	−2	−26	−45
CV/PLA 80:20	−23	−3	−15	−37
CV/PLA 70:30	−21	−11	−13	−39
Jute/PLA 90:10	−20	3	−14	−29
Jute/PLA 80:20	−45	2	−8	−48
Jute/PLA 70:30	−28	−3	−6	−34
Hemp/PLA 80:20	−34	0	−8	−39
Agro foil	−96	5	0	−96

Where—denotes decrease.

**Table 10:** The elongation at break change (%) of mulches in CD during winter field exposure.

Days	0–60	60–120	120–180	0–180
CV/PLA 90:10	11	−8	−6	−4
CV/PLA 80:20	−1	0	−6	−7
CV/PLA 70:30	−7	−8	−4	−17
Jute/PLA 90:10	−20	8	−21	−32
Jute/PLA 80:20	−28	2	−6	−31
Jute/PLA 70:30	−5	−4	−3	−11
Hemp/PLA 80:20	−25	−2	−7	−32
Agro foil	−8	9	−3	−3

Where—denotes decrease.

**Table 11:** The statistical analysis of the mulches’ elongation at break in MD and CD.

Samples Tested	*p*-Value
All nonwoven mulches and agro foil in MD	2.97 × 10^−12^
All nonwoven mulches in MD	1.80 × 10^−10^
All viscose nonwoven mulches in MD	2.92 × 10^−4^
All jute nonwoven mulches in MD	2.16 × 10^−4^
Viscose, jute, and hemp nonwoven mulch blends (80:20) in MD	1.08 × 10^−3^
Jute and hemp nonwoven mulch blends (80:20) in MD	0.03
All nonwoven mulches and agro foil in CD	3.30 × 10^−11^
All nonwoven mulches in CD	1.73 × 10^−11^
All viscose nonwoven mulches in CD	2.43 × 10^−6^
All jute nonwoven mulches in CD	5.47 × 10^−5^
Viscose, jute, and hemp nonwoven mulch blends (80:20) in CD	1.78 × 10^−3^
Jute and hemp nonwoven mulch blends (80:20) in CD	0.07

Where MD stands for machine direction, CD is the cross-machine direction.

**Table 12:** Soil temperature (°C) beneath nonwoven mulches and on the control field in winter 2022 and 2023.

Year	2022					2023																			
Month	Nov	December				January				February				March					April				May		
Date	17	2	8	16	22	5	12	20	27	1	9	17	22	3	9	14	24	30	6	12	21	27	4	11	19
CV/PLA																									
90:10	10.1	5.7	6.0	3.3	3.4	5.6	5.1	2.6	1.9	1.6	1.0	2.5	6.6	5.4	7.9	8.6	11.2	10.1	8.6	9.6	11.2	12.5	14.9	14.8	14.2
80:20	9.9	5.4	5.7	3.2	3.2	5.6	5.1	2.8	2.3	1.9	0.9	2.6	6.5	5.4	7.9	8.7	11.2	10.2	8.5	9.7	10.9	12.2	14.9	14.9	14.1
70:30	9.6	5.5	5.8	3.0	3.2	5.5	5.2	2.6	2.0	1.8	0.9	2.4	6.5	5.5	8.2	8.6	11.3	10.1	8.5	9.6	10.9	12.4	15.4	14.9	14.1
Jute/PLA																									
90:10	9.9	5.6	6.0	3.3	3.4	5.7	5.3	2.8	2.1	2.0	0.9	2.9	7.1	5.5	8.2	8.9	11.6	10.3	8.7	9.8	11.2	12.6	15.2	14.9	14.1
80:20	9.9	6.0	6.1	3.5	3.4	5.8	5.4	2.9	2.1	1.9	0.9	2.8	7.0	5.6	8.4	9.0	11.7	10.3	8.6	9.6	11.3	12.5	15.3	14.9	14.2
70:30	10.2	5.9	6.1	3.4	3.5	5.7	5.5	2.8	2.2	1.8	1.0	2.6	6.6	5.6	8.2	8.7	11.2	10.2	9.0	9.9	11.3	12.5	14.9	14.9	14.2
Hemp/PLA																									
80:20	10.0	5.8	6.2	3.4	3.5	10.5	5.4	2.8	2.1	1.9	1.1	2.9	6.8	5.5	8.3	8.9	11.7	10.5	8.8	9.9	11.3	12.7	15.1	14.9	14.3
Agro foil	9.9	5.7	6.2	3.6	3.5	5.9	5.7	3.0	2.1	2.2	0.9	3.2	7.0	5.5	8.6	9.4	12.0	10.8	8.9	10.0	11.5	12.8	15.3	15.0	14.2
CF	9.4	5.3	5.7	3.0	3.1	5.4	5.7	2.6	1.6	1.6	0.8	3.0	7.4	5.7	8.8	9.2	11.8	10.2	7.9	9.8	11.2	12.9	15.4	14.9	14.1
AT. (°C)	7.2	2.8	4.2	2.8	3.0	5.3	5.6	−0.4	0.4	0.8	−2.5	9.2	7.3	4.5	11.3	12.0	14.4	12.6	3.6	11.5	13.9	11.8	14.9	11.0	12.9
RH, %	98	97	97	99	96	94	96	98	99	98	97	93	96	97	93	95	91	94	95	93	89	92	92	99	97

Where AT is the average air temperature in °C; RH is relative air humidity %.

**Table 13:** Soil moisture (%) beneath nonwoven mulches and on the control field in winter 2022 and 2023.

Year	2022					2023																			
Month	Nov	December				January				February				March					April				May		
Date	17	2	8	16	22	5	12	20	27	1	9	17	22	3	9	14	24	30	6	12	21	27	4	11	19
CV/PLA																									
90:10	23.2	22.9	21.6	20.4	19.4	20.4	20.4	20.9	18.0	17.1	17.4	18.7	18.1	18.7	19.9	21.3	20.5	23.1	19.5	18.8	21.6	25.5	22.4	25.3	24.2
80:20	23.6	22.3	21.6	20.2	18.5	19.6	21.3	19.2	17.1	17.4	15.0	17.3	17.5	18.6	19.1	20.0	20.5	21.6	19.3	16.8	22.4	28.9	23.0	24.0	23.3
70:30	23.0	22.3	21.8	20.6	17.9	19.6	20.9	18.9	16.7	16.1	14.7	16.5	17.4	18.3	18.1	20.2	20.0	22.2	19.0	17.7	22.2	27.3	23.6	24.6	23.4
Jute/PLA																									
90:10	24.1	22.2	21.6	19.9	18.5	19.2	21.2	20.5	17.4	18.1	17.7	16.2	16.8	18.0	18.8	20.4	20.8	21.9	20.2	17.8	22.4	26.0	22.5	23.4	24.1
80:20	23.3	22.5	21.2	20.7	31.9	19.2	21.3	21.0	18.0	19.2	16.7	17.7	16.9	18.7	19.4	20.9	21.0	23.4	19.6	19.0	22.8	26.8	22.7	23.1	24.4
70:30	22.3	21.6	21.2	20.2	18.3	20.1	20.6	20.1	18.2	17.4	17.2	17.4	16.7	18.6	18.8	19.7	20.3	22.4	19.2	17.7	21.8	27.5	22.5	23.0	23.1
Hemp/PLA																									
80:20	23.5	22.2	21.0	20.2	18.9	19.2	21.3	19.9	17.5	17.4	15.7	16.9	16.5	19.8	18.9	21.0	20.1	22.0	20.0	17.7	21.2	27.6	22.1	25.2	23.6
Agro foil	23.4	21.2	21.4	19.5	18.6	18.9	20.7	20.3	17.3	17.7	15.0	17.8	18.3	19.7	19.3	20.8	21.3	22.7	20.3	18.2	21.9	28.3	21.6	23.6	23.7
CF	23.5	24.0	21.9	19.1	17.2	17.8	20.6	18.0	15.2	16.1	14.6	15.2	15.9	17.9	18.7	19.4	20.5	21.9	18.4	15.7	20.4	28.0	21.2	24.6	23.0
RH, %	98	97	97	99	96	94	96	98	99	98	97	93	96	97	93	95	91	94	95	93	89	92	92	99	97
AP, %	10.8	4.6	4.9	14.2	0	0	0.8	4.2	0.5	0	0	0	0	0	0	0	0	0	0	0	0	0	0.4	7.2	0.4

Where AP is the average precipitation in %; RH is relative air humidity %.

**Table 14:** Duncan statistical analysis of average soil temperature beneath the mulches and on the control field during winter experiment from November 2022 to May 2023.

Samples	2022/2023
CV/PLA 90:10	7.46 bc
CV/PLA 80:20	7.39 c
CV/PLA 70:30	7.41 bc
Jute/PLA 90:10	7.58 bc
Jute/PLA 80:20	7.60 bc
Jute/PLA 70:30	7.62 bc
Hemp/PLA 80:20	8.22 bc
Agro foil, kg/m^3^	7.87 ab

Different letters indicate significant differences according to the Duncan test (*p* ≤ 0.05).

**Table 15:** Soil quality analysis.

	pH		Humus, %	Organic C, %	Total N, %	P_2_O_5_, mg/100 g	K_2_O, mg/100 g
	H_2_O	1 MKCl					
Before experiment	7.85	7.08	4.30	2.91	0.29	5.40	28.07
CV/PLA 90:10	7.76	7.11	4.63	2.69	0.44	5.71	37.67
CV/PLA 80:20	7.80	7.16	4.01	2.33	0.48	4.18	37.00
CV/PLA 70:30	7.81	7.14	3.74	2.17	0.42	4.46	44.00
Jute/PLA 90:10	7.75	7.12	4.56	2.64	0.51	3.55	40.33
Jute/PLA 80:20	7.77	7.12	4.11	2.38	0.46	4.56	32.33
Jute/PLA 70:30	7.82	7.15	4.24	2.46	0.47	3.80	37.33
Hemp/PLA 80:20	7.71	7.11	4.63	2.68	0.39	4.84	41.67
Agro foil, kg/m^3^	7.78	7.14	4.34	2.51	0.43	3.55	42.00
Control field	7.81	7.13	4.11	2.38	0.28	4.95	31.00

**Table 16:** The weediness (%) on the control field and beneath mulches in the period from November 2022 to May 2023.

Samples		2023		
		January	March	May
CV/PLA	90:10, %	-	-	-
	80:20, %	-	-	-
	70:30, %	-	-	1.5
Jute/PLA	90:10, %	-	-	0.2
	80:20, %	-	-	0.4
	70:30, %	-	-	3.2
Hemp/PLA	80:20, %	-	-	0.4
Agro foil, %		-	-	0.1
Control field, %		100	100	100

## Data Availability

Data are contained within the article.
